# Rice Bran Wax-Based Matrix Tablets for Sustained Release of Diclofenac Sodium: Effects of Processing and Sintering

**DOI:** 10.3390/pharmaceutics18080936

**Published:** 2026-07-30

**Authors:** Nisit Kittipongpatana, Chawis Kingkaew, Pitsanu Duangkartok, Ornanong S. Kittipongpatana

**Affiliations:** 1 Department of Pharmaceutical Sciences, Faculty of Pharmacy, Chiang Mai University, Chiang Mai 50200, Thailand; nisit.k@cmu.ac.th; 2 Undergraduate Student in Pharm D. (Pharmaceutical Science) Program, Faculty of Pharmacy, Chiang Mai University, Chiang Mai 50200, Thailand; chawis_kingkaew@cmu.ac.th (C.K.); pitsanu_duangkartok@cmu.ac.th (P.D.)

**Keywords:** sustained release, diclofenac sodium, matrix tablets, melt granulation, rice bran wax, dissolution

## Abstract

**Background/Objectives:** Rice bran wax (RBW), a natural hydrophobic byproduct of rice bran oil refining, was evaluated as a lipid matrix former for the sustained oral delivery of diclofenac sodium (DFS). **Methods:** Matrix tablets containing 100 mg DFS and 45–60% (*w*/*w*) RBW, corresponding to 180–240 mg RBW per 400 mg tablet, were prepared using five techniques: simple mixing, dry granulation, extrusion–spheronization, partial melt granulation, and melt granulation. These techniques were selected to represent progressively different thermal, mechanical, and solvent-processing histories, ranging from simple physical blending to high-shear wet processing and extensive distribution of molten wax. **Results:** Processing method, wax concentration, and thermal sintering significantly influenced matrix structure and drug release. Scanning electron microscopy of the processed powders and granules indicated that melt granulation produced more extensively integrated wax-containing structures, although the internal continuity of the tablet matrix was not directly examined. FTIR and DSC analyses showed no evidence of major drug–excipient interactions and confirmed the retention of a detectable RBW melting transition. Melt granulation produced the greatest release retardation, followed by partial melt granulation, while extrusion–spheronization and dry granulation showed broadly similar release-retarding performance, and simple mixing was the least effective. The optimized formulation, containing 55% (*w*/*w*) RBW, prepared by melt granulation and sintered at 80 °C for 2 h, showed sustained release consistent with diffusion through the hydrophobic matrix. It met the dissolution limits specified in the USP–NF monograph for Diclofenac Sodium Extended-Release Tablets and showed a dissolution profile that was broadly comparable to that of the commercial reference product based on descriptive profile analysis. During a preliminary 4-week accelerated stability study, no statistically significant changes were observed in the physicochemical properties subjected to inferential analysis, while friability remained below 1%. The 55MG2 formulation also retained a broadly comparable dissolution profile, although a modest increase in drug release was observed. **Conclusions:** These findings support the potential of RBW as a natural lipid excipient for controlled oral drug delivery.

## 1. Introduction

Sustained release oral dosage forms are widely employed to improve therapeutic efficacy, minimize dosing frequency, and enhance patient adherence, particularly for drugs with short half-lives or dose-dependent adverse effects [1]. Diclofenac sodium (DFS), a nonsteroidal anti-inflammatory drug (NSAID), is commonly formulated as a sustained release matrix tablet to maintain stable plasma concentrations and reduce gastrointestinal irritation associated with rapid absorption [2]. Conventional sustained release formulations frequently rely on synthetic polymers such as hydroxypropyl methylcellulose (HPMC), ethyl cellulose, or acrylic copolymers [3,4,5]; however, increasing interest in naturally derived, sustainable excipients has encouraged the exploration of alternative matrix formers with hydrophobic or structuring properties capable of modulating drug release [2,6].

Rice bran wax (RBW) is a natural hard wax obtained as a by-product of rice bran oil refining and consists mainly of long-chain esters (C44–C64), fatty alcohols (C24–C28), and hydrocarbons. Its relatively high melting point (78–82 °C), low polarity, and strong hydrophobicity [7] make it a promising lipid-based barrier for limiting water penetration and controlling drug diffusion in matrix systems. RBW can retard drug release by forming a poorly wettable, recrystallized lipid network around drug and excipient particles. This network limits the penetration of the dissolution medium, increases the tortuosity of aqueous diffusion pathways, and thereby slows the dissolution and diffusion of diclofenac sodium from the tablet matrix. While lipid excipients such as carnauba wax, cetyl alcohol, stearic acid, and glycerides are widely used for sustained drug release [8,9,10], the pharmaceutical application of RBW as a matrix former remains relatively limited despite its biocompatibility, availability, low cost, and compatibility with melt-based processing. Owing to its relatively high melting point and ability to form hydrophobic structures upon cooling, RBW can function as an oleogel structuring agent or as a hydrophobic matrix-forming excipient.

The performance of hydrophobic matrix systems is strongly influenced by the type and concentration of lipid excipients, the drug–matrix composition, and the formulation process, which collectively affect matrix hydrophobicity, interparticle bonding, and the diffusion pathways governing drug release [11,12]. Techniques such as melt granulation, partial melt granulation, dry granulation, and extrusion–spheronization can modify wax morphology, particle–particle bonding, and matrix integrity, thereby altering the rate and mechanism of drug release [13,14]. Additionally, thermal treatment or sintering may promote wax mobility, redistribution, and interparticle fusion, thereby providing a potential approach for modifying matrix structure and fine-tuning release kinetics [15,16].

Although various lipid-based excipients have been investigated for controlled-release formulations, limited information is available regarding the application of RBW as a sustained release matrix former. The novelty of the present work lies not merely in demonstrating the release-retarding properties of a hydrophobic wax but in providing one of the few systematic evaluations of RBW for sustained release diclofenac sodium matrix tablets. In addition, the study directly compares five pharmaceutical processing methods, investigates the influence of thermal sintering on matrix performance, evaluates the association between measured tablet porosity and drug-release behavior, and identifies an RBW concentration that provides dissolution profiles meeting the limits specified in the USP–NF monograph for Diclofenac Sodium Extended-Release Tablets. Building upon our previous work that explored the application of RBW as a tablet lubricant [17], the present study aimed to further extend its utilization by developing and evaluating RBW-based matrix tablets of diclofenac sodium. The formulations were prepared using five distinct techniques—simple mixing, dry granulation, extrusion–spheronization, partial melt granulation, and melt granulation. The physicochemical properties and dissolution profiles were subsequently assessed, and the dissolution results were compared with the limits specified in the USP–NF monograph for Diclofenac Sodium Extended-Release Tablets to assess the potential of RBW as a natural matrix-forming excipient for controlled oral drug delivery.

## 2. Materials and Methods

### 2.1. Materials

Diclofenac sodium (DFS) was kindly supplied by Bangkok Lab and Cosmetic Co. Ltd. (Ratchaburi, Thailand) and used as the model drug. DFS was selected because it is a clinically relevant high-dose drug whose ionization and high apparent solubility in phosphate buffer at pH 7.5 create a demanding test for a hydrophobic wax matrix. Rice bran wax (RBW) was obtained from Koster Keunen (Watertown, CT, USA). A single supplier lot was used throughout the study. The material was not isolated directly from a specified rice cultivar or agricultural source. SMCC (Prosolv^®^; JRS Pharma, Rosenberg, Germany) was used as a co-processed direct-compression excipient. Magnesium stearate (MGS; Union Science Chemical, Chiang Mai, Thailand) served as a lubricant. Analytical-grade dipotassium hydrogen phosphate (K_2_HPO_4_) and potassium dihydrogen phosphate (KH_2_PO_4_) were used to prepare phosphate buffer (pH 7.5). Polyvinylpyrrolidone K90 (PVP K90; BASF SE, Ludwigshafen, Germany) was employed as the binder for extrusion–spheronization. Ethanol (95% *v*/*v*), distilled water, hydrochloric acid (HCl), and sodium hydroxide (NaOH) were used for granulation, pH adjustment, and dissolution media preparation. All other reagents were of analytical grade.

### 2.2. Tablet Preparation Methods

The formulations were composed of diclofenac sodium (DFS, 100 mg), rice bran wax (RBW), silicified microcrystalline cellulose (SMCC), and magnesium stearate (MGS), as shown in Table 1. Formulation codes were assigned according to RBW concentration, preparation method, and sintering duration. The initial number indicates the RBW content (% *w*/*w*), the letters indicate the preparation method—SM, simple mixing; DG, dry granulation; ES, extrusion–spheronization; PMG, partial melt granulation; and MG, melt granulation—and the final numeral indicates the sintering duration at 80 °C (1 = 1 h and 2 = 2 h). For example, 55MG2 denotes a formulation containing 55% (*w*/*w*) RBW, prepared by melt granulation and sintered for 2 h.

RBW concentrations of 45–60% (*w*/*w*), corresponding to 180–240 mg per 400 mg tablet, were selected as a practical formulation-screening range. The lower boundary was chosen to provide a sufficiently high hydrophobic-matrix fraction for sustained release, whereas the upper boundary preserved an adequate proportion of SMCC for powder consolidation and tablet manufacture. Higher RBW contents were not pursued because further displacement of SMCC was expected to compromise compactability and increase the likelihood of excessive release retardation. All ingredients were accurately weighed using an analytical balance (Mettler Toledo, Greifensee, Switzerland). Each formulation batch consisted of 40 g of blended powder, equivalent to 100 tablets at the target weight of 400 mg. Tablets were compressed using a hydraulic tablet press (Carver Inc., Wabash, IN, USA) equipped with 10.3 mm flat-faced punches. A compression force of 9.81 kN was applied and maintained for approximately 10 s prior to decompression. Only internal lubrication with magnesium stearate was used; no external die-wall lubricant was applied, and no sticking, picking or capping was observed under the selected conditions. Granulation was performed using an oscillating granulator fitted with a No. 16 mesh screen. Thermal processing, including drying and sintering, was conducted in a hot-air oven. The sintering temperature of 80 °C was selected to promote softening and interparticle fusion of RBW without complete melting or loss of tablet geometry. Tablets were placed on a flat tray without an applied load during heating and were allowed to cool to room temperature before handling. No collapse, warping, or gross deformation was observed after sintering. RBW was melted in a temperature-controlled water bath. Extrusion–spheronization studies were carried out using a Caleva Multi Lab Classic (Model MLC-230, Caleva Process Solutions Ltd., Sturminster Newton, Dorset, UK). The five preparation methods were selected to provide contrasting thermal, mechanical, solvent, and particle-consolidation histories that could influence the distribution and organization of RBW within the formulation and the resulting tablet properties. Simple mixing served as the least-processed control, in which the components were combined without granulation or melting. Dry granulation represented a solvent-free process involving pre-compression and mechanical size reduction. Extrusion–spheronization represented a wet, high-shear pelletization process involving binder-assisted agglomeration and subsequent drying. Partial melt granulation involved incorporation of DFS into molten RBW before the addition of SMCC, thereby producing localized wax-mediated agglomeration. Melt granulation involved complete mixing of DFS and SMCC with molten RBW, allowing broader redistribution of the wax throughout the formulation. Together, these methods were used to examine how differences in thermal exposure, shear, solvent use, and wax distribution affected matrix structure and drug-release behavior. For the process-comparison experiment, tablets prepared by all five methods were subjected to the same post-compression sintering treatment at 80 °C for 1 h. No unsintered simple-mixing tablets were included in this comparison; therefore, this experiment was designed to compare preparation methods under a common thermal-treatment condition rather than to isolate the effect of sintering in the simple-mixing formulation.

#### 2.2.1. Simple Mixing (SM)

DFS, RBW and SMCC powders were weighed and mixed by tumbling in a plastic bag until homogeneous. MGS was then added, and the powder blend was compressed. The compressed tablets were subjected to the common process-comparison sintering condition of 80 °C for 1 h; no unsintered SM control was evaluated.

#### 2.2.2. Dry Granulation (DG)

DFS and RBW were dry-mixed and pre-compressed using 25 mm punches at 2.94 kN. The larger punch size facilitated the preparation of slugs for subsequent milling through a No. 16 mesh, while the low compression force produced coherent but friable slugs suitable for granulation. MGS was added, followed by a short mixing. The final blend was compressed and sintered at 80 °C for 1 h.

#### 2.2.3. Extrusion–Spheronization (ES)

DFS and RBW were mixed and moistened with a 5% (*w*/*v*) PVP K90 solution prepared in a 1:1 mixture of 95% ethanol and water, corresponding to approximately 47.5% (*v*/*v*) ethanol. For each 40 g batch, 16 mL of the binder solution was added, providing 0.8 g PVP K90, equivalent to 2% (*w*/*w*) of the dry formulation. PVP K90 was used only in the extrusion–spheronization formulations and replaced an equivalent amount of SMCC so that the total dry batch weight and target tablet weight remained unchanged. The PVP K90 concentration was kept constant across all ES formulations. The wet mass was extruded and spheronized after brief solvent exposure at ambient temperature, and the resulting pellets were dried at 60 °C for 1 h. The hydroalcoholic vehicle may have transiently softened surface lipid fractions of RBW and contributed to agglomeration, although this effect was not independently evaluated. SMCC was then added and mixed thoroughly, followed by MGS and a short final mixing step. The final blend was compressed into tablets and sintered at 80 °C for 1 h.

#### 2.2.4. Partial Melt Granulation (PMG)

RBW was melted over a water bath, and DFS was incorporated with continuous and thorough stirring, then allowed to cool down under ambient laboratory conditions at 28 ± 2 °C for 15 min without forced cooling. The solidified homogeneous mass was sieved through a no. 16 mesh to obtain granules. SMCC was then added and mixed thoroughly. MGS was added, followed by a short mixing step. The final blend was compressed and sintered at 80 °C for 1 h.

#### 2.2.5. Melt Granulation (MG)

RBW was melted, after which DFS and SMCC were added and mixed continuously until a homogeneous mixture was obtained. The molten mass was maintained at approximately 80–85 °C, and DFS exposure to the molten wax was limited to the time required for mixing and spreading. The mixture was spread into a thin layer and cooled under ambient laboratory conditions at 28 ± 2 °C for 15 min without forced cooling. The solidified mass was then passed through a No. 16 mesh sieve, briefly mixed with MGS, compressed into tablets, and sintered at 80 °C for 1 h.

#### 2.2.6. Effect of Sintering Time

Based on the overall experimental design, melt granulation (MG) tablets were additionally prepared using the procedure described in Section 2.2.5, except that the sintering time at 80 °C was extended from 1 h to 2 h to investigate the effect of prolonged sintering.

### 2.3. Physicochemical Evaluation of Sample Powder and Tablets

#### 2.3.1. Scanning Electron Microscopy (SEM)

The morphological characteristics of the samples were examined using a CLARA field-emission scanning electron microscope (FESEM; TESCAN, Brno, Czech Republic). Samples were pre-dried at 60 °C for 24 h to remove residual moisture. This temperature was below the measured melting onset of RBW. The samples were mounted on adhesive tape attached to copper stubs and sputter-coated with gold under vacuum using a CCU-010 coating system (Safematic GmbH, Zizers, Switzerland). SEM imaging was performed at an accelerating voltage of 10 kV. Images were acquired from representative regions using short acquisition times to minimize potential beam-induced alterations.

#### 2.3.2. Fourier Transform Infrared (FTIR) Spectroscopy

FTIR spectra were recorded using an INVENIO S spectrophotometer (Bruker, Billerica, MA, USA). Samples were pre-dried at 60 °C for 24 h and analyzed directly in attenuated total reflectance (ATR) mode to assess potential chemical changes resulting from the melting/granulation process.

#### 2.3.3. Differential Scanning Calorimetry (DSC)

The thermal properties of the samples were obtained using a DSC 8000 differential scanning calorimeter (PerkinElmer Inc., Waltham, MA, USA). Samples were analyzed from 30 to 350 °C at a heating rate of 10 °C/min. The thermal transition parameters, including onset temperature (To), peak temperature (Tp), end temperature (Te), and enthalpy change (ΔH), were evaluated.

#### 2.3.4. Tablet Porosity

Tablet porosity was calculated using the density method from the tablet bulk density and the true density of the powder blend, as previously described for the determination of nominal tablet porosity [18]. The tablet bulk (apparent) density was determined from tablet mass and geometric volume, whereas the true (skeletal) density (ρ_true_) of the powder blend was measured using an AccuPyc II 1340 helium gas pycnometer (Micromeritics, Norcross, GA, USA) at 28 ± 2 °C. Each measurement was repeated ten times. Tablet porosity (ε) was computed according to:

$$\varepsilon (\%)=\left(1|\frac{\rho_{\mathrm{bulk}}}{\rho_{\mathrm{true}}}\right)\times 100$$

where $\rho_{\mathrm{bulk}}$ is the bulk density (g/cm^3^) and $\rho_{\mathrm{true}}$ is the true (skeletal) density (g/cm^3^).

### 2.4. Standard Quality Control of Tablets

#### 2.4.1. Tablet Hardness

Ten (10) tablets were randomly selected and evaluated using a tablet hardness tester (PTB-311, Pharma Test Apparatebau AG, Hainburg, Germany). The breaking force was recorded for each tablet, and the mean hardness and standard deviation (SD) were calculated.

#### 2.4.2. Friability

Twenty (20) tablets were dedusted and accurately weighed. The tablets were placed in a friability tester (PTF 20E, Pharmatest, Germany) and rotated at 25 rpm for 4 min (100 revolutions). After 100 revolutions, the tablets were removed, dedusted, and reweighed. Friability was calculated from the percentage loss in total tablet mass, thereby accounting for abrasion and edge rounding.

$$\%\mathrm{Friability}=\frac{\mathrm{Initial}\;\mathrm{weight}-\mathrm{Final}\;\mathrm{weight}}{\mathrm{Initial}\;\mathrm{weight}}\times 100$$

A weight loss of not more than 1.0% was considered acceptable. Any tablet exhibiting cracking, cleavage, capping, or breakage was considered to have failed the test regardless of the calculated percentage weight loss.

#### 2.4.3. Tablet Thickness

Tablet thickness was measured individually using a tablet thickness gauge (OXD3300100K, Oxford Precision, Wigston, UK). Ten tablets (n = 10) were evaluated. The mean values and standard deviation (SD) were recorded.

### 2.5. Drug Dissolution and Profile Comparison

#### 2.5.1. Dissolution Study

Dissolution testing was conducted using USP Apparatus II with paddles rotating at 50 rpm. Each vessel contained 900 mL of 0.05 M phosphate buffer at pH 7.5, maintained at 37 ± 0.5 °C. This single-stage dissolution medium was selected in accordance with the USP–NF monograph for Diclofenac Sodium Extended-Release Tablets to permit direct compendial assessment and comparison. The test was not intended to simulate the sequential pH conditions of the human gastrointestinal tract. Aliquots of 10 mL were withdrawn at 1, 5, 10, 16, and 24 h for analysis. These sampling times were selected to correspond to the specified time points in the USP–NF monograph for Diclofenac Sodium Extended-Release Tablets and were primarily intended for compendial dissolution profile assessment rather than detailed characterization of the early burst phase. An equal volume of fresh medium was added to maintain the overall volume.

#### 2.5.2. Quantification of DFS in Samples

Samples withdrawn at predetermined time intervals during the dissolution study were immediately filtered through a 0.45 µm nylon syringe filter, appropriately diluted, and analyzed using a double-beam UV-2450 UV–Visible spectrophotometer (Shimadzu Corporation, Kyoto, Japan) at a wavelength of 276 nm. Drug concentrations were determined from a previously constructed calibration curve. Filter-recovery validation was not performed; therefore, complete recovery of DFS through the nylon membrane could not be confirmed.

The amount of diclofenac sodium released at each sampling time point was calculated as follows:

(a) Concentration of diluted sample

$$x_{\mathrm{diluted}}=\frac{y-b}{a}$$

where y = measured absorbance, a = slope of calibration curve, and b = intercept of calibration curve.

(b) Concentration in dissolution vessel

$$C_{n}=x_{\mathrm{diluted}}\times DF$$

where C_n_ = actual drug concentration in the dissolution vessel at time point n, DF = dilution factor, and Dilution factor (DF) = final assay volume ÷ aliquot volume.

(c) Amount of drug in the dissolution vessel

$$M_{\mathrm{vessel},n}=V\times C_{n}$$

where M_vessel,n_ = amount of drug in the vessel at time point n (mg), V = dissolution medium volume (900 mL), C_n_ = drug concentration at time point n.

(d) Cumulative amount of drug released

$$M_{\mathrm{cum},n}=M_{\mathrm{vessel},n}+\sum_{i=1}^{n-1}(V_{s}\cdot C_{i})$$

where M_cum,n_ = cumulative amount of drug released at time point n (mg), Vs = sample withdrawal volume (10 mL), and Ci = drug concentration at previous sampling time points.

This equation corrects for drug loss due to sample withdrawal during the dissolution test.

(e) Percentage cumulative drug release

$${\%D}_{n}=\frac{M_{\mathrm{cum},n}}{M_{0}}\times 100$$

where %Dn = cumulative percentage of drug released at time point n, M_cum,n_ = cumulative amount release, and M_0_ = nominal amount of drug per tablet.

### 2.6. Preliminary Short-Term Accelerated Stability Study

Based on the sintering optimization study, the 55MG2 formulation was selected for a preliminary short-term accelerated stability study. Tablets were stored at 40 ± 2 °C/75 ± 5% RH for 4 weeks. At the end of storage, the tablets were evaluated for changes in appearance, hardness, friability, porosity, and dissolution profile and compared with those of freshly prepared tablets (Week 0). This short-term study was intended to identify preliminary changes under accelerated conditions and was not designed to establish product shelf life.

### 2.7. Dissolution Profile Analysis

#### 2.7.1. Model-Independent Dissolution Profile Comparison

Dissolution profiles were compared using the model-independent difference factor ($f_{1}$) and similarity factor ($f_{2}$) [19]. The commercial diclofenac sodium extended-release tablet was designated as the reference product, while the freshly prepared 55MG2 formulation and the 55MG2 formulation after 4 weeks of accelerated storage were designated as test products. Mean cumulative drug-release values obtained at 1, 5, 10, 16, and 24 h were used for the calculations.

The difference factor was calculated as:

$$f_{1}=\left[\frac{\sum_{t=1}^{n}|R_{t}-T_{t}|}{\sum_{t=1}^{n}R_{t}}\right]\times 100$$

and the similarity factor was calculated as:

$$f_{2}=50\log_{10}\left\{{\left[1+\frac{1}{n}\sum_{t=1}^{n}(R_{t}-T_{t}{)}^{2}\right]}^{-0.5}\times 100\right\}$$

where $R_{t}$ and $T_{t}$ represent the mean percentages of drug released from the reference and test products, respectively, at time point t, and n is the number of dissolution sampling time points.

Pairwise comparisons were performed between the commercial reference and 55MG2 at Week 0, between the commercial reference and 55MG2 after 4 weeks of accelerated storage, and between 55MG2 at Week 0 and Week 4. The comparisons with the commercial product were used to assess agreement with the reference-release profile, whereas the Week 0–Week 4 comparison was used to evaluate dissolution profile stability.

For descriptive interpretation, $f_{1}$ values of 0–15 and $f_{2}$ values of 50–100 were taken as indicating close profile agreement. Because the dissolution experiments were conducted in triplicate and the analysis was not designed as a formal regulatory similarity assessment, the calculated values were interpreted descriptively rather than as evidence of regulatory dissolution equivalence.

#### 2.7.2. Weibull Modeling of Dissolution Profiles

The complete 24 h dissolution profiles of the commercial diclofenac sodium extended-release product, freshly prepared 55MG2 tablets at Week 0, and 55MG2 tablets after 4 weeks of accelerated storage were additionally evaluated using nonlinear Weibull modeling. Each replicate dissolution profile was fitted separately using nonlinear least-squares regression according to the two-parameter Weibull equation:

$$F\left(t\right)=100\left[1-\exp\left(-{\left(\frac{t}{\alpha }\right)}^{\beta }\right)\right]$$

where $F\left(t\right)$ is the cumulative percentage of drug released at time t, α is the Weibull scale parameter, and β is the Weibull shape parameter.

The asymptotic maximum release was fixed at 100%, and lag time was not estimated because only five dissolution sampling times were available. This reduced model was selected to minimize overparameterization. The time required for 50% drug release ($t_{50}$) was calculated as:

$$t_{50}=\alpha {\left(\ln2\right)}^{1/\beta }$$

Model performance was evaluated using the coefficient of determination ($R^{2}$), root-mean-square error (RMSE), and visual agreement between the observed and model-predicted dissolution values. Weibull parameters obtained from the three independently fitted replicate profiles were summarized as mean ± standard deviation.

The Weibull model was used as an empirical descriptor of dissolution profile shape and release time scale. Its parameters were not interpreted as definitive evidence of a specific physicochemical drug-release mechanism [20,21,22].

### 2.8. Statistical Analysis

Unless otherwise specified, experiments were conducted in triplicate, and results are reported as mean ± standard deviation. Statistical analyses were performed using SPSS version 19.0. Differences among more than two groups were assessed by one-way analysis of variance followed by Tukey’s honestly significant difference test, with statistical significance set at p<0.05. Friability was measured as a single pooled-tablet determination for each formulation and was not subjected to inferential statistical analysis.

## 3. Results and Discussion

### 3.1. Physicochemical Evaluation of Powder and Tablets

#### 3.1.1. Scanning Electron Microscopy (SEM)

**Figure 1:**
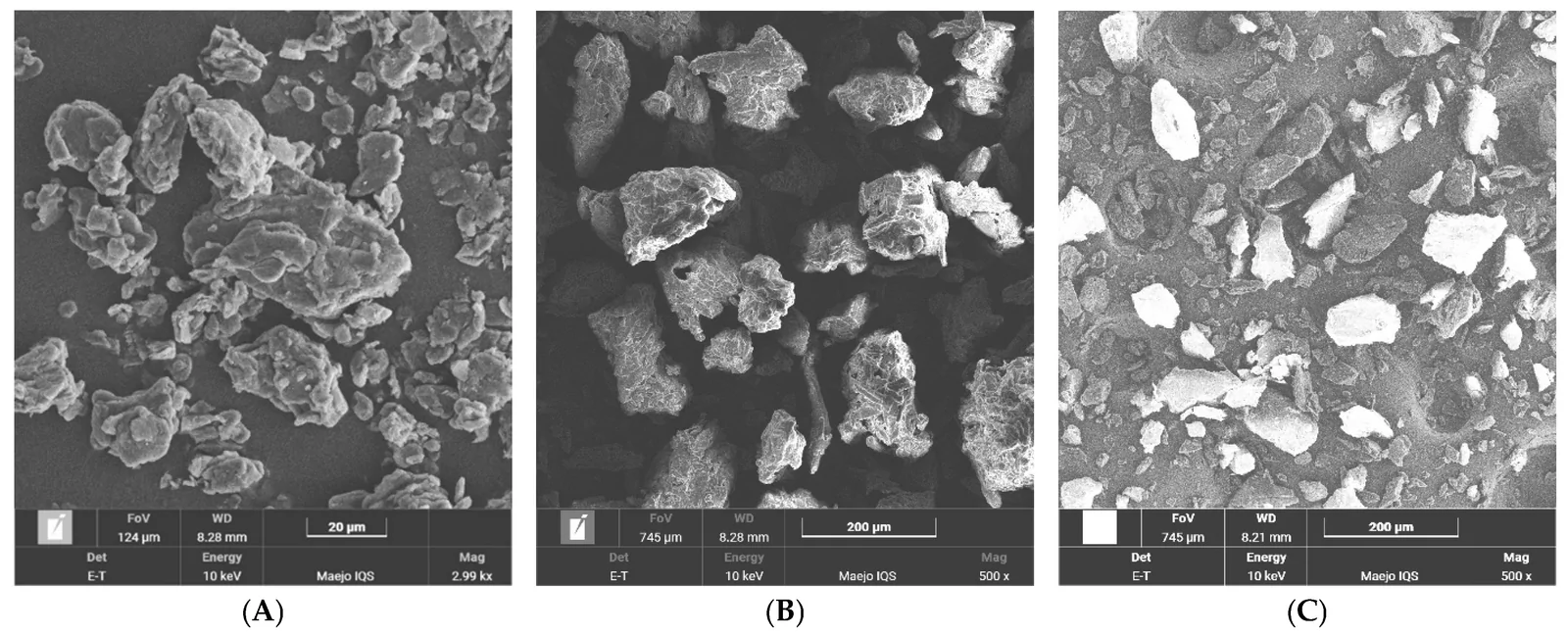
SEM images of (**A**) diclofenac powder (2000×), (**B**) SMCC powder (500×), and (**C**) RBW (500×).

Based on scanning electron microscopy (SEM) images at 2000× magnification, diclofenac powder exhibited thin, plate-like crystals of irregular small size with rough and fractured surfaces and noticeable agglomeration (Figure 1A). The sharp edges and angular morphology were consistent with the particulate appearance of the drug. The particles showed a broad apparent size range; however, packing density and interparticle void structure were not directly evaluated. SEM images of SMCC at 500× magnification revealed irregular particles with sharp edges and a broad particle size distribution (Figure 1B). This morphology is consistent with that commonly reported for cellulose-based excipients and may be associated with water uptake and penetration of the dissolution medium [11]. However, the wicking rate and water uptake were not directly measured in this study. The SEM images of RBW at 500× magnification showed irregular fractured aggregates with rough and glossy surfaces (Figure 1C), consistent with the reported morphology of a hydrophobic wax material [17]. These observations are compatible with the role of RBW as a hydrophobic matrix-forming excipient; however, wettability and internal porosity cannot be determined directly from SEM images alone. The SEM observations were limited to particle morphology and were not intended to characterize the flow or packing properties of the raw RBW powder. Because lipid-based materials may undergo localized deformation or other beam-induced changes during electron microscopy, the SEM images were interpreted qualitatively and may not fully represent the native, unexposed microstructure of the samples.

**Figure 2:**
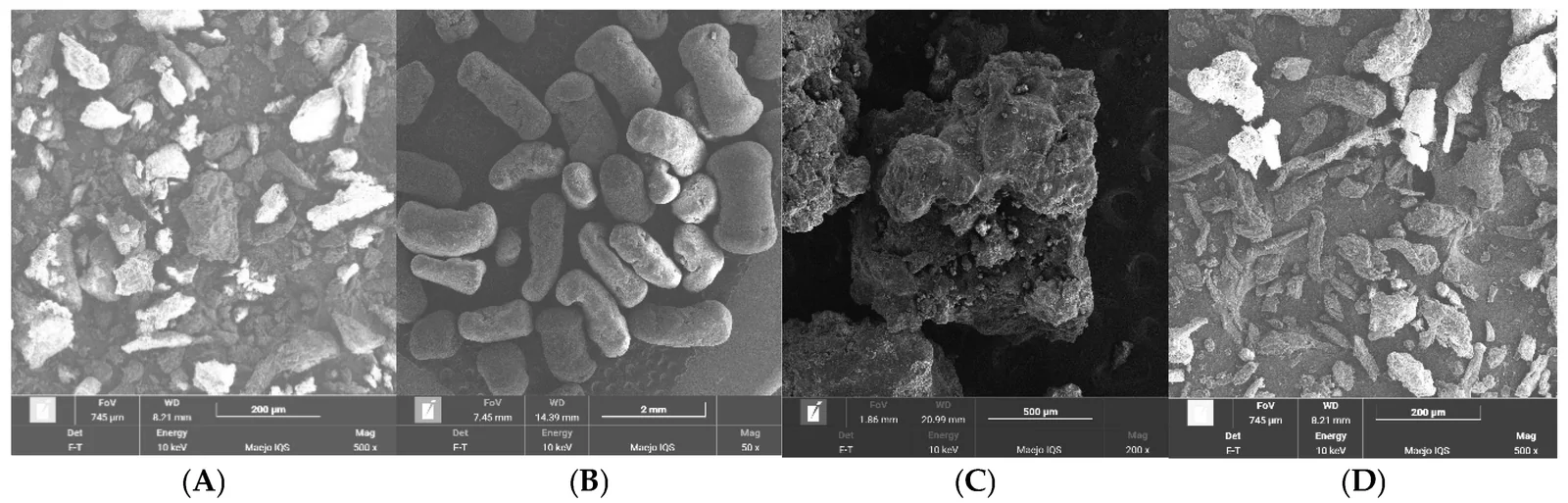
SEM images of processed powders or granule formulations prepared by (**A**) simple mixing (SM), (**B**) extrusion–spheronization (ES), (**C**) melt granulation (MG), and (**D**) partial melt granulation (PMG).

Figure 2 shows SEM images revealing distinct morphological differences among the controlled-release powder formulations. The SM formulation exhibited irregular, angular particles with rough surfaces and a wide size distribution, suggesting a simple physical mixture with limited visible particle fusion. ES showed more regularly shaped particles with smooth to moderately rough surfaces. Minor surface irregularities and partial rounding were consistent with the spheronization process, although the distribution of RBW within the pellets could not be determined directly from the SEM images. In contrast, the MG formulation displayed compacted agglomerated clusters with partially fused surfaces and fewer visible interparticle gaps, suggesting more extensive wax-mediated agglomeration. The PMG formulation demonstrated intermediate morphological characteristics, with partial agglomeration and moderate visible cohesion but less extensive fusion than the MG formulation. Overall, the progression from relatively discrete particles in the SM and ES formulations to more agglomerated structures in the MG formulation was consistent with greater agglomeration of the processed granules and may contribute to the observed drug-release behavior. Because cross-sectional SEM images of the compressed and sintered tablets were not obtained, these observations are limited to the surface morphology of the processed granules/powders and do not directly demonstrate the internal continuity of the tablet matrix.

#### 3.1.2. FTIR Analysis and Compatibility Study

**Figure 3:**
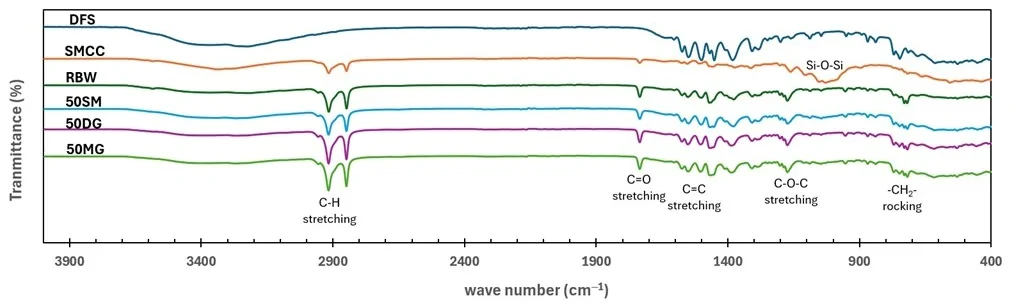
FTIR spectra of pure DFS, SMCC, RBW and the formulation containing 50% RBW prepared by simple mixing (SM), dry granulation (DG), and melt granulation (MG).

The FTIR spectra of DFS, SMCC, RBW, and the 50% wax formulation prepared by the SM, DG, and MG methods are presented in Figure 3. DFS exhibited characteristic aromatic-ring C=C stretching bands around 1600–1500 cm^−1^ [23], together with bands attributable to the ionized carboxylate group of diclofenac sodium. These spectral features were consistent with the sodium salt form of the drug. The SMCC spectrum showed a broad O–H stretching band at approximately 3400 cm^−1^, typical of hydrogen-bonded hydroxyl groups in cellulose, and C–H stretching near 2900 cm^−1^. Strong absorption bands in the 1100–1000 cm^−1^ region correspond to C–O–C stretching vibrations of the β-1,4-glycosidic linkage. A band attributable to Si–O–Si stretching at 1050–1080 cm^−1^ was consistent with the presence of colloidal silicon dioxide in SMCC. RBW demonstrated intense symmetric and asymmetric C–H stretching bands at approximately 2915 and 2848 cm^−1^ and a distinct ester carbonyl band near 1730 cm^−1^. The carbonyl band observed in RBW-containing formulations was assigned primarily to the ester groups of RBW rather than to the free carboxylic acid form of diclofenac. No clear FTIR evidence of the conversion of diclofenac sodium to diclofenac free acid was observed under the processing conditions studied.

The spectra of formulations prepared by the simple mixing (SM), dry granulation (DG), and melt granulation (MG) methods retained the principal characteristic absorption bands of DFS, SMCC, and RBW. Minor differences in peak intensity and partial band overlap were observed; however, no new absorption bands or major peak shifts were detected. These findings provided no clear FTIR evidence of a major chemical interaction between DFS and the excipients under the processing conditions examined. The observed spectral variations were likely due to dilution effects, differences in component distribution, and overlapping bands arising from the presence and distribution of DFS and the excipients within the formulation. Nevertheless, FTIR is not a stability-indicating technique and cannot exclude minor degradation products. It also cannot definitively determine whether DFS was present in a crystalline or molecularly dispersed state within the wax matrix.

#### 3.1.3. Differential Scanning Calorimetry (DSC)

**Figure 4:**
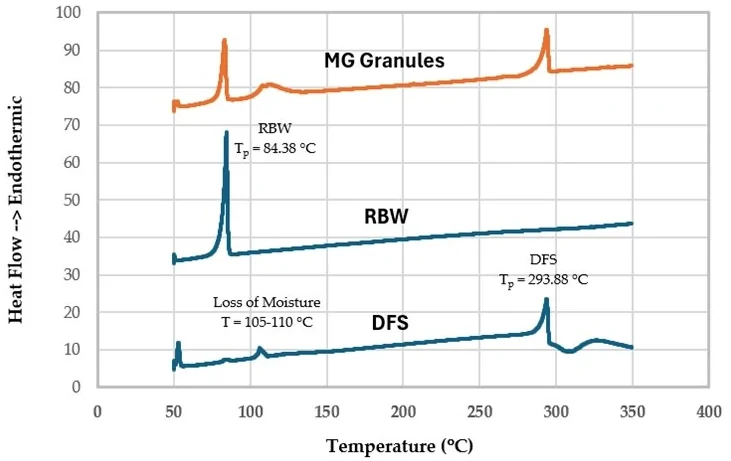
DSC thermograms of pure DFS, RBW and a representative formulation containing 50% RBW prepared by melt granulation (MG).

Differential scanning calorimetry (DSC) was performed to investigate the thermal behavior and physical state of diclofenac sodium (DFS), rice bran wax (RBW), and the melt granulation formulation containing 50% (*w*/*w*) RBW and 25% (*w*/*w*) DFS. The DSC thermograms are presented in Figure 4.

The DSC thermogram of pure RBW exhibited a single, sharp endothermic melting peak with an onset temperature of 82.52 °C, a peak temperature of 84.38 °C, and an endset temperature of 85.56 °C (Figure 4). The melting enthalpy (ΔH) was 213.39 J/g. Accordingly, the oven set point of 80 °C used for tablet sintering was approximately 2.5 °C below the measured melting onset of the RBW lot used in this study. The treatment was therefore intended to increase wax mobility and promote interparticle fusion while avoiding complete bulk melting and structural collapse of the tablets.

The narrow melting range and relatively high enthalpy are consistent with substantial crystalline organization within the RBW sample; however, DSC alone cannot establish crystal homogeneity, crystal-size distribution, or polymorphic form. This thermal behavior is consistent with the long-chain wax-ester composition of RBW and its tendency to undergo crystalline organization upon cooling. Dassanayake et al. reported melting temperatures of bulk RBW between 77 and 79 °C, while other studies have reported melting ranges extending to approximately 82–86 °C, depending on the wax source, purification process, chemical composition, and DSC operating conditions [24]. Therefore, the melting peak observed in the present study falls within the reported range. The slightly higher melting temperature and enthalpy compared with some literature reports may reflect differences in material source, wax-ester composition, purification history, sample preparation, or DSC operating conditions.

The DSC thermogram of pure DFS exhibited a well-defined thermal event with an onset temperature of 290.00 °C, peak temperature of 293.88 °C, and endset temperature of 295.36 °C. The corresponding melting enthalpy was 104.95 J/g. The well-defined thermal transition was consistent with the presence of crystalline DFS. The observed transition temperature agrees well with previously reported DSC studies of crystalline diclofenac sodium, which exhibits a major thermal event around 290–295 °C [25]. It should be noted, however, that this transition represents melting accompanied by thermal decomposition rather than a simple melting process, as diclofenac sodium undergoes degradation immediately following melting at elevated temperatures. Consequently, the observed peak is more appropriately described as the characteristic melting/decomposition-related thermal transition of crystalline DFS.

The DSC thermogram of the melt granulation formulation retained two distinct thermal transitions corresponding to both formulation components. The first endothermic peak appeared at approximately 84–85 °C, corresponding to the melting of RBW, while the second thermal event remained at approximately 294–295 °C, corresponding to the characteristic melting/decomposition transition of DFS. Compared with the pure materials, both peaks exhibited lower intensity, which is expected because the individual components were diluted within the formulation. In addition, the RBW melting peak appeared slightly broader than that of the pure wax, suggesting altered thermal organization of the wax matrix following melt granulation. Such peak broadening has been reported in lipid-based formulations and may be associated with recrystallization of the molten lipid and the presence of dispersed drug particles within the wax matrix [26]. However, DSC alone cannot determine crystal-size distribution or confirm polymorphic or crystallographic changes. PXRD analysis would be required to establish whether processing induced such structural transitions. Importantly, the characteristic thermal transition of DFS remained clearly detectable in the formulation with no substantial shift in peak temperature. The persistence of this thermal event indicates that a substantial proportion of DFS remained in its crystalline state following melt granulation. If DFS had been completely dissolved in the molten wax and subsequently converted into an amorphous solid dispersion or molecularly dispersed within the lipid matrix, the characteristic crystalline thermal transition would be expected to disappear or become markedly diminished or shifted [1]. Instead, the simultaneous observation of both the RBW melting peak and the DFS melting/decomposition peak suggests that the formulation retained detectable thermal transitions attributable to both DFS and RBW, consistent with the persistence of crystalline fractions of both components after melt granulation. However, retention of the DFS transition does not exclude minor thermal degradation.

#### 3.1.4. Tablet Porosity

The porosity of DFS–SMCC–RBW matrix tablets ranged from 9.69% to 13.63% (Table 2). These values are within the range reported for compressed hydrophobic matrix tablets and reflected differences in the apparent degree of consolidation among formulations [20,21]. The highest porosity was observed for 45SM1 (13.63 ± 0.35%), whereas 60PMG1 exhibited the lowest value (9.69 ± 0.34%). Increasing RBW concentration generally reduced tablet porosity, although the magnitude of this effect depended on the preparation method. The trend was most apparent for simple-mixed formulations, where porosity decreased from 13.63% at 45% RBW to 10.73% at 60% RBW. Similar but less pronounced reductions were observed for dry-granulated, extrusion-spheronized, partial melt-granulated, and melt-granulated systems. The decrease in porosity with increasing wax content may reflect changes in particle packing and compact structure associated with the greater wax fraction, although the underlying consolidation mechanism was not directly evaluated. The preparation method exerted a substantial influence on tablet porosity. At equivalent RBW concentrations, simple-mixed formulations generally exhibited the highest porosity, while dry granulation, partial melt granulation, and melt granulation produced denser matrices with porosity values typically ranging from approximately 10–11%. In contrast, extrusion–spheronization formulations consistently exhibited relatively high porosity (11.45–11.71%) across all RBW concentrations. This observation suggests that the granule architecture generated during extrusion–spheronization was retained after compression, limiting the extent of particle rearrangement and densification compared with the other preparation techniques. Statistical analysis demonstrated significant differences among selected formulations (*p* < 0.05).

### 3.2. Standard Quality Control of Tablets

Tablet hardness ranged from 69.5 to 102.6 N, with the highest values obtained from the PMG and MG methods at 45% RBW (100.4 ± 2.7 and 102.6 ± 2.5 N, respectively). In general, tablet hardness decreased as the RBW content increased to 60%, especially for the SM (75.4 ± 4.2 N), PMG (71.7 ± 5.9 N), and MG (69.5 ± 3.0 N) formulations. Tablet friability ranged from 0.37 to 3.57%. Tablets prepared by DG, ES, PMG, and MG showed low friability (0.37–1.21%), while SM tablets had much higher friability (1.78–3.57%), exceeding the pharmacopeial limit of 1%. The high friability of the SM formulations was likely associated with inadequate interparticle consolidation. RBW has previously been shown to possess tablet-lubricant functionality [17]. At the high wax concentrations used in the present study, discretely distributed RBW particles may have exerted a lubricant-like interfacial effect that reduced bonding between DFS and SMCC during compression. In the absence of granulation or melting to redistribute and integrate the wax within the matrix, this effect may have contributed to weaker compacts and greater susceptibility to abrasion, although the mechanism was not directly evaluated. Consequently, despite its operational simplicity, the SM method was considered unsuitable for producing commercially robust tablets under the tested conditions. The PMG tablets at 60% RBW showed a friability of 0.44%, whereas the MG tablets exhibited the lowest friability (0.37%), indicating high resistance to abrasion despite having the lowest hardness among all formulations. Under a constant compression force (9.81 kN) and a fixed die diameter, tablet thickness ranged from 3.89 to 4.50 mm, reflecting formulation-dependent differences in compaction behavior and post-compression recovery.

The mechanical properties of the DFS tablets were closely related to tablet porosity. In several formulations, lower porosity was associated with higher hardness and lower friability; however, this relationship was not consistent at the highest RBW concentration. The MG method, which produced the best controlled-release performance, also generated tablets with among the lowest porosity values (10.20–10.98%) and consistently low friability. These results suggest that the MG produced tablets with relatively low measured porosity and low friability. In contrast, the SM method had the highest porosity (10.73–13.63%) and inadequate mechanical strength, consistent with its elevated friability. Although increasing the RBW content generally reduced porosity, hardness did not continue to increase at 60% RBW. For example, MG and PMG tablets became softer even though their porosity remained low. This suggests that a high amount of RBW reduced the strength of interparticle bonding because the larger wax fraction deformed more easily during compression. However, the wax-rich matrix retained low friability despite reduced hardness.

Visual examination after sintering showed that the tablets retained their overall shape and structural integrity, with no gross deformation, collapse, or visible surface melting. Because all tablets were compressed under the same nominal conditions, differences in thickness may reflect formulation-dependent variations in compaction and elastic recovery. However, thickness alone could not explain tablet hardness at high RBW contents. Measured porosity and the organization of the hydrophobic wax-containing matrix may have contributed to drug release by influencing resistance to liquid ingress and drug transport, although water penetration and internal matrix structure were not directly measured. Tablet hardness primarily reflects the mechanical strength of interparticle bonding and should not be regarded as a direct indicator of release-controlling performance.

### 3.3. Drug Release

#### 3.3.1. Influence of Preparation Method

**Figure 5:**
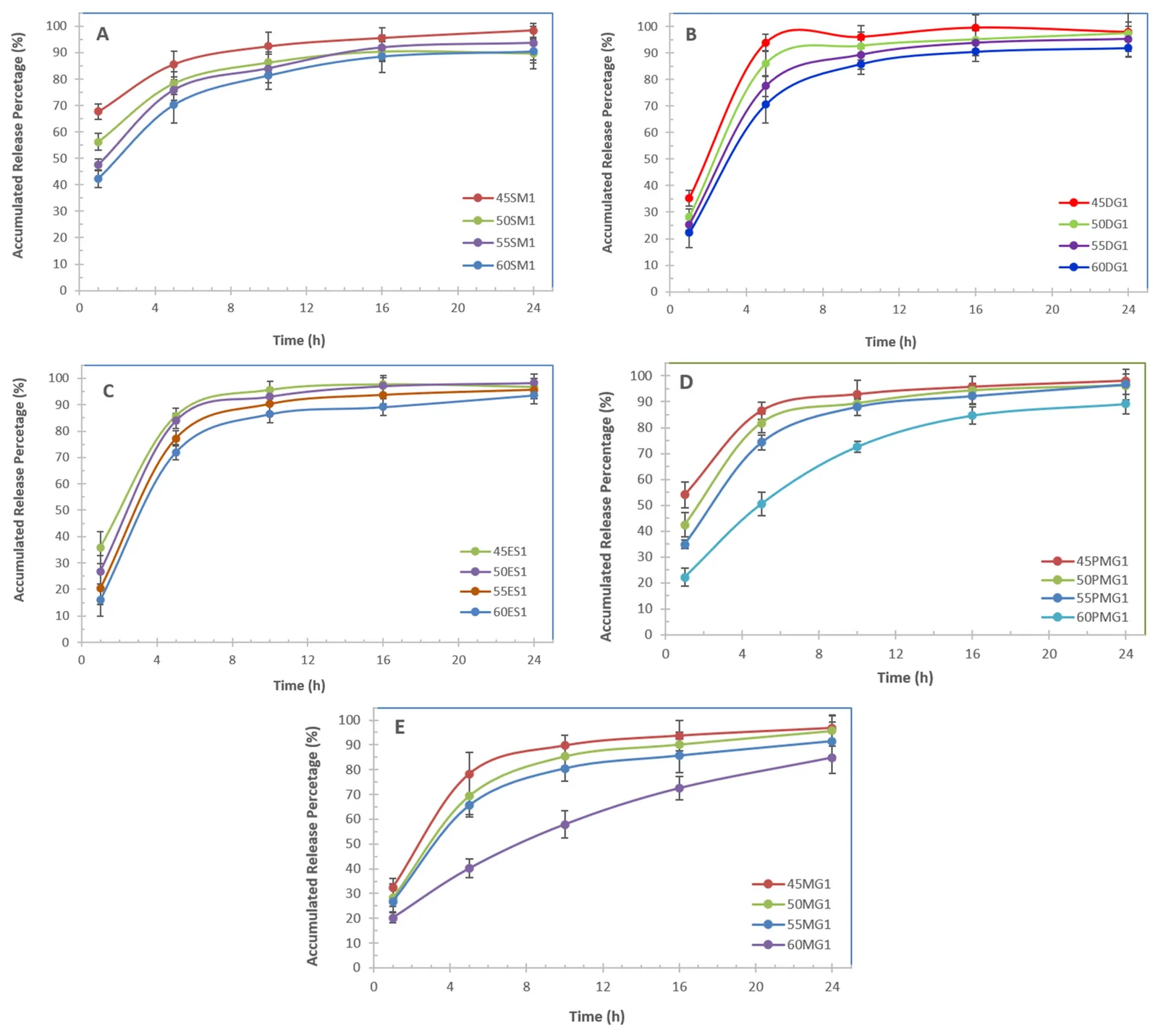
Dissolution profiles of diclofenac sodium (DFS) matrix tablets prepared by (**A**) simple mixing (SM), (**B**) dry granulation (DG), (**C**) extrusion–spheronization (ES), (**D**) partial melt granulation (PMG), and (**E**) melt granulation (MG). Formulations containing 45–60% (*w*/*w*) rice bran wax (RBW) were initially prepared using a 1 h sintering process (80 °C) and evaluated to identify the most suitable preparation method.

The dissolution behavior of diclofenac sodium was strongly influenced by the preparation technique. Distinct release profiles were observed among the simple mixing (SM), dry granulation (DG), extrusion–spheronization (ES), melt granulation (MG), and partial melt granulation (PMG) systems, all of which sintered at 80 °C for 1 h and evaluated over 24 h (Figure 5A–E). Overall, the preparation method had a marked influence on drug release in addition to formulation composition, highlighting the importance of matrix formation during processing. This finding is consistent with previous work showing that different processing methods applied to the same wax-matrix composition produced distinct matrix structures and dissolution profiles [27].

Among the five preparation methods, the SM formulations exhibited the fastest drug release, characterized by a pronounced initial burst and rapid dissolution throughout the study. The absence of a granulation or melting step likely resulted in less effective distribution of RBW within the powder mixture, which may have produced a less effective hydrophobic barrier; however, medium penetration and internal diffusion pathways were not directly measured. The DG and ES formulations provided improved control of the initial burst release compared with SM, although their overall dissolution profiles remained relatively rapid. The granulation process enhanced particle consolidation and matrix integrity, reducing early drug release; however, the predominantly particulate distribution of wax was insufficient to produce release retardation comparable to that of the melt-based formulations. The PMG formulations exhibited intermediate release behavior between the conventional granulation methods and the fully melt-based system. Partial melting promoted greater redistribution of RBW than DG or ES, resulting in slower drug release. Nevertheless, because SMCC was incorporated after the wax–drug mass had cooled and solidified, wax redistribution throughout the complete formulation was likely less extensive than in melt granulation, leading to less effective diffusion control. The MG formulations demonstrated the greatest ability to sustain diclofenac release and produced dissolution profiles that most closely resembled those of the commercial sustained release product. A similar trend has been reported for Compritol-based wax matrix tablets, where melt granulation and partial melt granulation produced slower drug release than dry blending or spray drying, reflecting differences in matrix structure generated during processing [27]. Melting RBW during granulation likely promoted more extensive wax redistribution and integration among formulation components after cooling. This more integrated wax-containing structure may have provided a more effective hydrophobic barrier and increased resistance to drug transport, thereby providing sustained drug release over the entire dissolution period. The release-retarding performance generally followed the order MG > PMG > ES/DG > SM. ES and DG showed broadly similar dissolution retardation under the tested conditions, despite their distinct particle structures, porosity, and mechanical properties. These findings indicate that ther manufacturing technique influences drug release through the combined effects of wax distribution, particle consolidation, porosity, and matrix structure rather than through any single physical parameter. The superior performance of melt granulation suggests that more extensive redistribution and integration of molten RBW contributed to the formation of a more effective hydrophobic diffusion barrier and improved sustained release behavior.

#### 3.3.2. Effect of Rice Bran Wax Percentage

Having established that the preparation method markedly influenced drug release, the effect of RBW concentration was evaluated within each preparation technique. In all systems, increasing RBW reduced the initial burst release and prolonged drug release, although the extent of the effect depended on the manufacturing method. The effect was smallest in the SM formulations. Increasing RBW reduced the 1 h release from approximately 68% (45SM1) to 42% (60SM1), but all formulations exceeded the USP upper limit (35%), indicating that simple physical mixing could not produce an effective hydrophobic matrix to control early drug release. A greater concentration-dependent effect was observed in the DG formulations. The 1 h release decreased from approximately 35% (45DG1) to 22% (60DG1), allowing formulations containing 50–60% RBW to meet the USP specification. However, all DG formulations exceeded the USP upper limit at 5 h, indicating that drug release remained too rapid despite the reduced initial burst. This suggests that increasing wax content alone could not compensate for the comparatively limited release-retarding structure produced by dry granulation. The ES formulations also showed slower drug release with increasing RBW content. The 1 h release decreased from approximately 36% (45ES1) to 27, 20 and 16% for 50ES1, 55ES1 and 60ES1, respectively, meeting the USP specification at this time point. However, all ES formulations exceeded the USP upper limit at 5 h, indicating that drug release remained too rapid thereafter despite the reduced initial burst. The relatively rapid later-stage release may partly be associated with the presence of PVP K90, which was used at a constant concentration of 2% *w*/*w* as a binder during extrusion–spheronization. Although PVP K90 provided the wet-mass cohesiveness and mechanical integrity required for extrusion and spheronization, its water solubility may allow it to progressively dissolve and leach from the matrix during dissolution. This process may generate additional aqueous pathways and increase the effective porosity of the wax matrix, thereby facilitating water penetration and drug diffusion over time and partially counteracting the release-retarding effect of RBW. However, PVP leaching and changes in matrix porosity were not directly characterized in the present study. Thus, although extrusion–spheronization produced a more homogeneous wax–drug matrix than SM or DG, the resulting matrix was still unable to sustain drug release within the desired USP profile.

The concentration-dependent effect was most pronounced in the MG system. Increasing RBW from 45% to 60% reduced drug release from approximately 33% to 20% at 1 h and from approximately 89% to 58% at 10 h. Formulations containing 45–55% RBW maintained sustained release characteristics while achieving nearly complete release by 24 h. Within the 1 h-sintered MG series, 60MG1 showed the slowest release profile, with its 10 h value approaching the lower limit of the USP target range, suggesting that excessive wax loading may over-restrict drug diffusion.

This trend should be interpreted as the combined effect of increasing the hydrophobic RBW fraction and simultaneously decreasing the SMCC fraction. Increasing the wax fraction may reduce matrix wettability and water penetration and increase resistance to drug transport, consistent with previous observations that greater wax loading decreases drug release from wax matrix tablets [9,11,28]. Conversely, the microcrystalline cellulose component of SMCC may promote water uptake and the formation of aqueous pathways, while its colloidal silicon dioxide component may influence powder packing and pore structure. Thus, reducing SMCC may further limit liquid penetration and hydrophilic-channel formation, reinforcing the release-retarding effect of higher RBW content.

The magnitude of this coupled compositional effect also depended on the manufacturing method. It was modest in the SM and DG formulations but more pronounced in the ES and especially the MG formulations, where more extensive wax redistribution and particle integration likely produced a more effective hydrophobic barrier. However, because RBW and SMCC were varied inversely rather than independently, the observed release trend cannot be attributed solely to RBW concentration.

#### 3.3.3. Effect of Sintering

Melt granulation was selected for further investigation because it produced the most promising dissolution performance among the preparation methods. To evaluate the effect of sintering duration, melt-granulated formulations containing 45–60% RBW were sintered at 80 °C for either 1 h (MG1) or 2 h (MG2), and the corresponding dissolution profiles were compared across the full concentration range.

**Figure 6:**
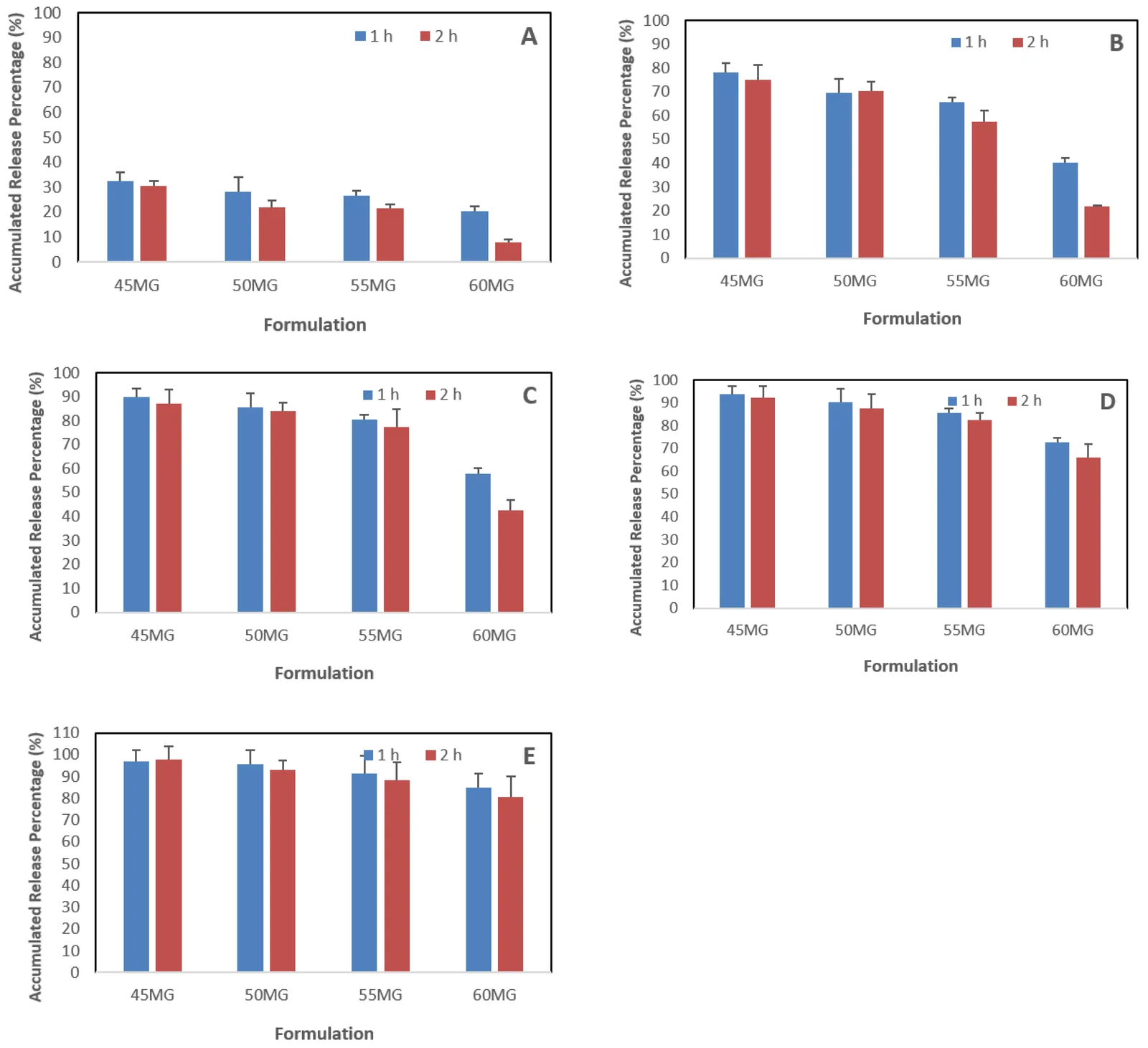
Effect of sintering time on the dissolution behavior of melt granulation (MG) formulations containing 45–60% (*w*/*w*) rice bran wax (RBW). Tablets were sintered at 80 °C for either 1 or 2 h, and cumulative drug release was compared at dissolution times of (**A**) 1 h, (**B**) 5 h, (**C**) 10 h, (**D**) 16 h, and (**E**) 24 h.

At the 1 h dissolution time point (Figure 6A), extending the sintering duration from 1 to 2 h generally reduced drug release. The 45MG formulation showed only a slight decrease, whereas the 50MG and 55MG formulations exhibited larger reductions. The greatest change occurred in the 60MG formulation, where drug release decreased from approximately 20% to 8%. A similar pattern was observed at 5 h (Figure 6B). Drug release from the 45MG and 50MG formulations changed only slightly after 2 h of sintering, whereas the 55MG formulation showed a moderate reduction. Again, the largest decrease was observed for the 60MG formulation, with release falling from approximately 40% to 22%. The same trend persisted at 10 and 16 h (Figure 6C,D), although the differences gradually became smaller as drug release approached completion. These findings show that prolonged sintering increased the release-retarding effect, particularly at higher RBW contents. This behavior may be associated with greater wax mobility, redistribution, or interparticle fusion during thermal treatment, resulting in a more effective hydrophobic barrier [29]. However, paired hardness and porosity measurements were not available for all MG1–MG2 formulations; therefore, increased tablet density or matrix consolidation cannot be confirmed directly from the present data.

Comparison of the sintering conditions demonstrated that extending the sintering time from 1 h to 2 h produced a dissolution profile that more closely matched the target release characteristics while remaining within the USP acceptance criteria. Therefore, the optimized formulations used for all subsequent investigations, including kinetic model analysis and accelerated stability testing, were prepared using the 2 h sintering condition (MG2), unless otherwise stated.

#### 3.3.4. Relationship with Tablet Porosity

Tablet porosity is an important structural attribute because it influences mass transport, liquid penetration, and drug dissolution within pharmaceutical tablets [18,30,31]. In wax matrix systems specifically, residual porosity and dissolution-induced pore formation can facilitate water penetration and create pathways for drug release [28]. In the present study, the SM formulations exhibited the highest porosity (10.73–13.63%), consistent with their rapid dissolution profiles, suggesting an association between higher measured porosity and faster drug release; however, pore connectivity and liquid penetration were not directly measured. In contrast, the PMG and MG formulations generally showed lower porosity (9.69–10.98%), consistent with their lower measured porosity and slower drug-release profiles that likely restricted solvent ingress and contributed to slower drug release. Within most preparation methods, increasing RBW concentration produced a gradual reduction in porosity, particularly in the SM and PMG formulations, whereas only minor changes were observed for the DG, ES, and MG systems. Interestingly, the ES formulations maintained relatively high porosity (11.45–11.71%) while exhibiting slower drug release than the DG formulations, demonstrating that tablet porosity alone cannot account for the observed dissolution behavior. Instead, differences in wax distribution, particle bonding, and overall matrix organization generated during processing are also important in controlling water penetration and drug diffusion. These findings indicate that sustained drug release resulted from the combined effects of tablet porosity and the organization of the hydrophobic wax-containing matrix. Consistent with the SEM observations, melt-based processing, particularly melt granulation, likely promoted more extensive wax redistribution and particle integration, producing a more effective diffusion barrier despite only modest differences in measured porosity. Therefore, tablet porosity is an important but not sufficient predictor of drug release and should be interpreted together with matrix microstructure.

#### 3.3.5. Comparison with USP Release Criteria

Comparison with the dissolution limits specified in the USP-NF monograph for Diclofenac Sodium Extended-Release Tablets (15–35% at 1 h; 45–65% at 5 h; 65–85% at 10 h; 75–95% at 16 h; and 80–100% at 24 h) [32] revealed clear differences among the preparation methods. The SM formulations exceeded the USP upper limits at both 1 and 5 h, indicating poor control of the initial burst release. The DG formulations met the 1 h criterion at higher RBW contents (50–60%) but exceeded the 5 h upper limit, demonstrating that increasing wax content alone could not adequately sustain drug release. The ES formulations also exceeded the 5 h upper limit despite meeting the 1 h criterion, indicating that although ES produced more regularly shaped particles in the SEM images (Figure 2), it was still unable to maintain the desired sustained release profile. In contrast, MG formulations showed the best overall agreement with the USP dissolution criteria.

**Figure 7:**
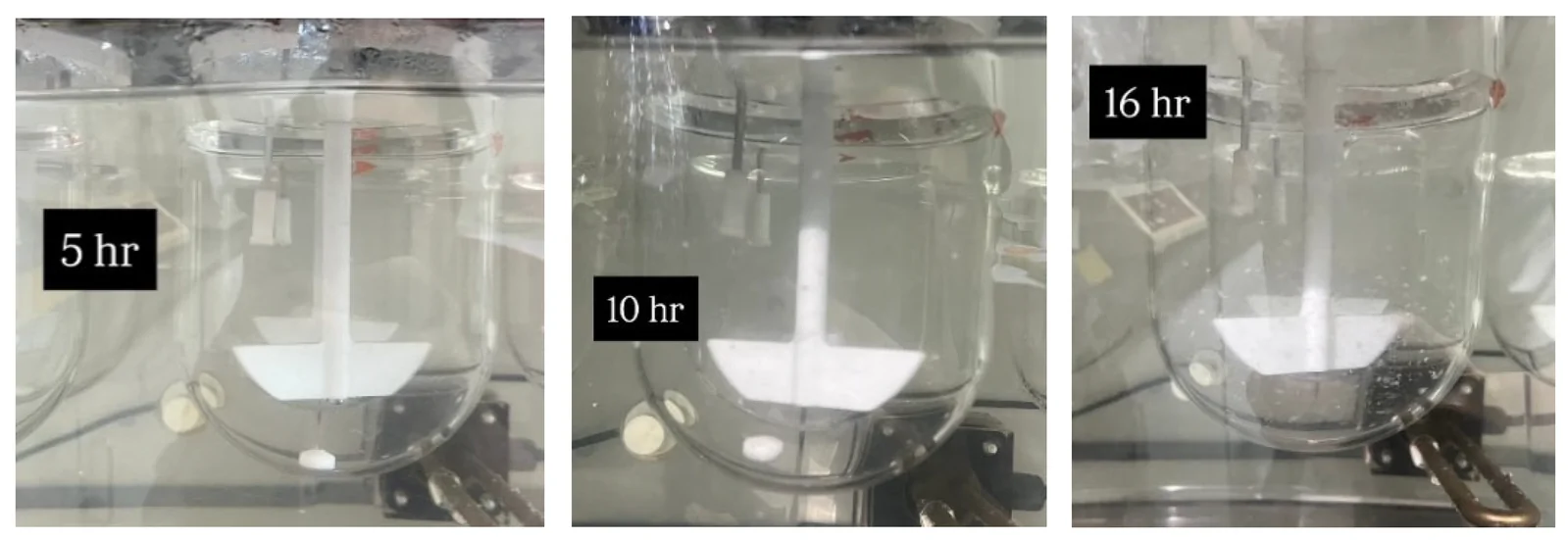
Visual observation of the integrity of the 55MG2 tablet during dissolution testing at 5, 10, and 16 h.

Among the MG2 formulations, 55MG2 met the USP dissolution criteria at all evaluated time points, whereas 60MG2 showed insufficient release at 1, 5, 10, and 16 h. The exact mean ± SD cumulative-release values for all MG2 formulations and the commercial reference are provided in Table 3. These findings indicate that, among the formulations evaluated, 55% RBW with 2 h of sintering provided the most appropriate balance between controlling the initial burst release and maintaining the target sustained release profile. Overall, melt granulation combined with 2 h of sintering produced the closest agreement with the USP sustained release dissolution profile, with 55MG2 providing the optimal balance between burst release control and sustained drug release. Visual observation during dissolution testing showed that the 55MG2 tablets retained their overall physical integrity at 5, 10, and 16 h (Figure 7). The enhanced performance is consistent with the SEM observations (Figure 2), which suggest greater matrix consolidation and wax coalescence following melt processing, together with the possible effects of prolonged thermal treatment on wax mobility, redistribution, and interparticle fusion [33,34].

#### 3.3.6. Release Kinetic Analysis of the Screening Formulations

The kinetic-fitting results are presented in Table 4. Because the kinetic models were evaluated using their conventional linearized forms, which involve different transformations of the release data, the resulting r^2^ values were interpreted only as descriptive measures of agreement with each individual model. Accordingly, comparisons of r^2^ values across models should not be regarded as definitive statistical evidence that one model provides a uniquely superior fit. For 45MG2, 50MG2, and 55MG2, the Higuchi equation produced relatively high r^2^ values. For 55MG2, the Higuchi equation gave an r^2^ value of 0.9788, indicating that the release profile was compatible with the mathematical relationship described by this model. This finding is consistent with diffusion contributing to drug release from the hydrophobic RBW matrix [34]. However, the kinetic results alone do not establish that diffusion was the sole or predominant release mechanism.

The Korsmeyer–Peppas model also showed relatively high $r^{2}$ values for all formulations. However, because the release exponent was not suitable for categorical mechanistic interpretation, these fits do not independently establish the contribution of matrix structural changes. Potential processes such as water uptake by hydrophilic excipients, pore development following drug dissolution, matrix relaxation, erosion, or fragmentation remain plausible hypotheses. Because water uptake, swelling, erosion, fragmentation, and changes in matrix porosity were not directly measured, the present results cannot confirm non-Fickian transport or a combined diffusion–erosion mechanism.

For 60MG2, high r^2^ values were observed with the Korsmeyer–Peppas and first-order models (r^2^ = 0.9913 and 0.9910, respectively), whereas the Higuchi equation produced a lower r^2^ value of 0.8763. This suggests that at higher wax content, drug release becomes more complex. These findings indicate that the release profile of 60MG2 differed in its mathematical agreement with the evaluated equations compared with the lower-RBW formulations. However, the small difference between the Korsmeyer–Peppas and first-order r^2^ values does not permit identification of a uniquely best-fitting model or a specific release mechanism.

RBW may act as a hydrophobic barrier that limits the penetration of the dissolution medium and the diffusion of dissolved drug through the matrix, whereas the hydrophilic properties of SMCC may promote interaction with the aqueous medium. Changes in matrix porosity, surface area, and diffusion pathways during dissolution can influence drug-release behavior [35]. However, water penetration, water uptake, swelling, SMCC redistribution, pore development, matrix relaxation, erosion, fragmentation, and other internal structural changes were not directly measured. Their contributions to drug release therefore remain plausible mechanistic hypotheses rather than experimentally demonstrated processes.

Thus, the observed release profiles were consistent with diffusion through the hydrophobic RBW-containing matrix, with possible contributions from progressive matrix structural changes; however, the available kinetic data were insufficient to establish a unique mechanism or confirm non-Fickian transport. Mechanistic interpretation was limited by fitting only five compendial sampling time points, which may not adequately characterize the early release phase or provide sufficient temporal resolution. Moreover, water uptake, swelling, dimensional changes, erosion, mass loss, fragmentation, and microstructural changes were not measured. Accordingly, the conventional linearized kinetic models were retained as descriptive assessments of the screening formulations rather than as definitive mechanistic classifications. The complete dissolution profiles of the selected 55MG2 formulation and the commercial reference product were subsequently evaluated using nonlinear Weibull modeling, as described in Section 3.3.7. Future studies should quantify matrix changes at multiple dissolution time points to distinguish the relative contributions of diffusion and structural modification during drug release.

#### 3.3.7. Weibull Analysis of the Selected Formulation

To provide a nonlinear evaluation of the complete 24 h dissolution profiles, the commercial diclofenac sodium extended-release product, freshly prepared 55MG2 tablets at Week 0, and 55MG2 tablets after 4 weeks of accelerated storage were fitted to the two-parameter Weibull model. The fitted parameters are presented in Table 5.

The commercial product showed a mean Weibull scale parameter (α) of 5.56±0.72 h and a mean shape parameter (β) of 0.79±0.05. The corresponding values for 55MG2 at Week 0 were 6.64±0.76 h and 0.70±0.07, respectively, whereas 55MG2 at Week 4 showed an α value of 5.43±0.87 h and a β value of 0.77±0.05.

The calculated median release times ($t_{50}$) were 3.49±0.38 h for the commercial product, 3.91±0.26 h for 55MG2 at Week 0, and 3.37±0.42 h for 55MG2 at Week 4. The larger α and longer $t_{50}$ observed for the freshly prepared 55MG2 tablets were consistent with their slightly slower observed drug-release profile. After 4 weeks of accelerated storage, both parameters decreased, corresponding to the modest increase in drug release observed experimentally.

The mean $R^{2}$ values were 0.988±0.007, 0.967±0.023, and 0.996±0.004 for the commercial product, 55MG2 Week 0, and 55MG2 Week 4, respectively. The corresponding RMSE values were 3.77±1.34, 5.52±2.21, and 1.78±0.98. These results indicated generally acceptable descriptive agreement between the observed and Weibull-predicted dissolution values.

All three profiles exhibited mean β values below 1, describing a curve with a relatively rapid initial phase followed by progressively slower release. However, because the first dissolution measurement was obtained at 1 h, the present dataset could not directly quantify or distinguish an initial surface-associated burst from subsequent release through the internal matrix. Moreover, because the Weibull model is empirical, its parameters were interpreted as descriptors of release-profile shape and time scale rather than as evidence of a specific physicochemical release mechanism.

### 3.4. Preliminary Short-Term Accelerated Stability Study and Optimal Formulation

Tablet formulation 55MG2 showed no visible changes in color, surface appearance, or physical integrity after 4 weeks of accelerated storage at 40 °C/75% RH. Tablet hardness decreased only slightly from 78.0 ± 1.2 N to 75.6 ± 2.0 N, while porosity remained statistically unchanged, with values of 8.89 ± 0.61% at Week 0 and 8.40 ± 0.45% after 4 weeks $\left(p>0.05\right)$. Tablet thickness remained unchanged (4.19 mm), and friability remained below 1% throughout the study, indicating no substantial deterioration in the measured mechanical properties during the 4-week study.

**Figure 8:**
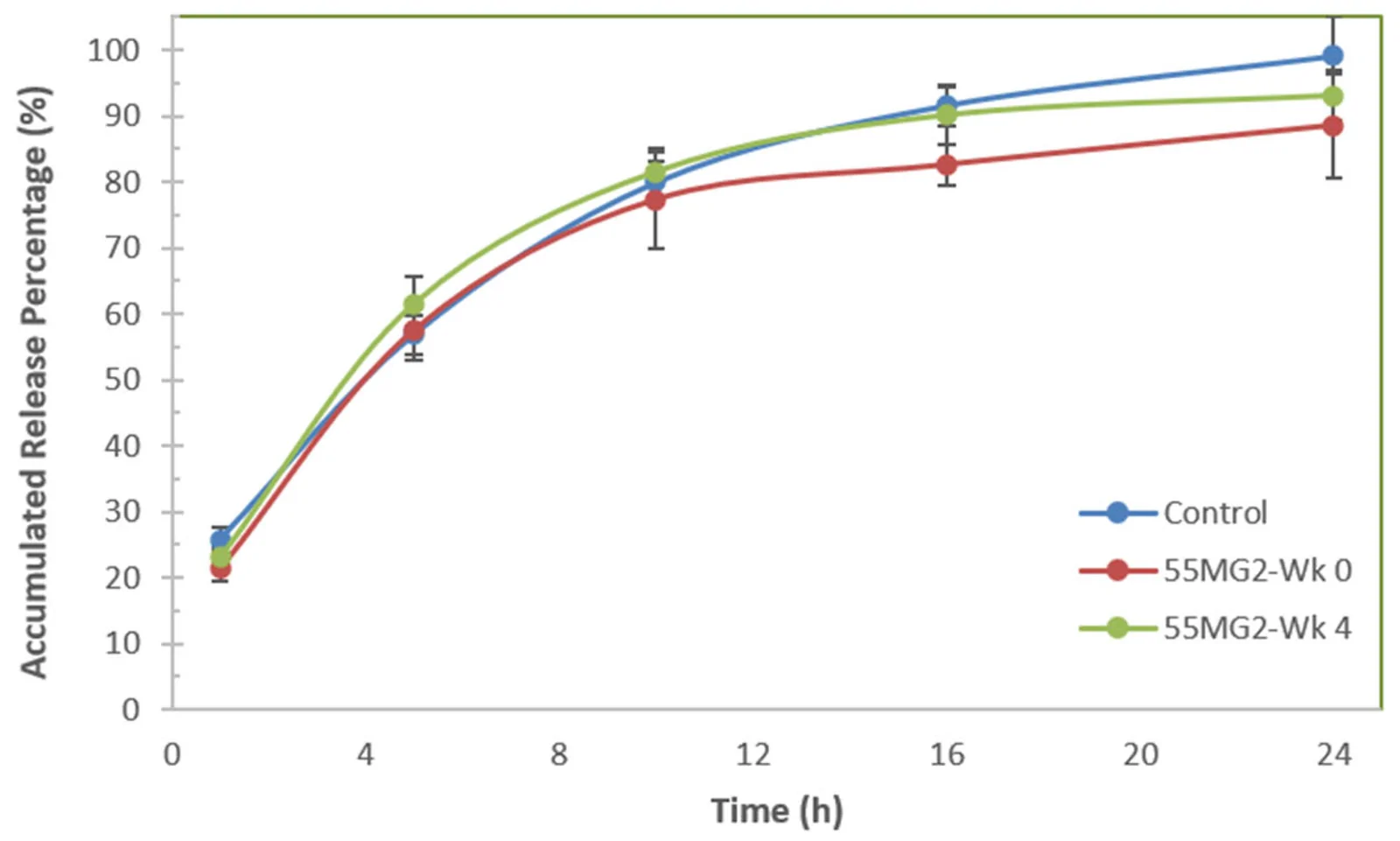
Dissolution profiles of a commercial diclofenac sodium extended-release tablet (Control), freshly prepared 55MG2 tablets (55% RBW, 2 h sintering), and 55MG2 tablets after accelerated stability testing (40 °C/75% RH for 4 weeks).

The dissolution profiles of the commercial product (control), freshly prepared 55MG2 tablets (Week 0), and tablets stored under accelerated conditions for 4 weeks (Week 4) are shown in Figure 8. The drug released at the 1 h sampling point may partly represent an initial burst caused by rapid dissolution of drug located at or near the tablet surface. Because no samples were collected before 1 h, the magnitude and duration of this early release phase could not be fully characterized. Future studies should therefore include earlier sampling points, such as 15, 30, and 45 min, to better differentiate surface drug dissolution from subsequent release governed by diffusion through and/or structural changes within the internal matrix.

The freshly prepared 55MG2 tablets exhibited slightly slower drug release than the commercial product throughout the dissolution period, reflecting a somewhat stronger sustained release effect. The Week 4 tablets showed slightly higher drug release than the freshly prepared tablets at several sampling times. Comparison of the Week 0 and Week 4 dissolution profiles yielded an *f*_1_ value of 6.81 and an *f*_2_ value of 65.40 (Table 6). Based on these values, the profiles were considered similar. The nonlinear Weibull analysis was consistent with this trend, as the scale parameter, α, and the median release time, t_50_, decreased after 4 weeks of accelerated storage and approached the values obtained for the commercial product, indicating a modest increase in the overall release rate. At 24 h, cumulative drug release increased from approximately 89% for the fresh tablets to 93% after storage, while the commercial product released approximately 99%.

The observed changes were small and did not suggest a marked loss of release control under the tested conditions. Overall, the 55MG2 formulation retained broadly comparable controlled-release characteristics and mechanical properties during the 4-week study. The formulation remained within an acceptable sustained release profile and showed no substantial changes during the 4-week preliminary study. Based on its dissolution performance, agreement with the USP-NF dissolution limits, and preliminary short-term stability findings, 55MG2 was selected as the optimal formulation for further development.

### 3.5. Study Limitations

This study was designed primarily as a formulation-screening and process-comparison investigation. Its main limitations include the absence of comprehensive RBW micromeritic and lot-to-lot characterization, an unsintered simple-mixing control, independent variation in RBW and SMCC, stability-indicating impurity analysis, filter-recovery validation, and direct measurements of water uptake, swelling, erosion, PVP leaching, and matrix microstructure. Cooling rate, crystallographic changes, and tablet cross-sectional morphology were also not characterized, while the five compendial dissolution time points provided limited resolution for mechanistic modeling.

In addition, the use of a single pH 7.5 dissolution medium and a 4-week accelerated stability study was intended for preliminary compendial and stability assessment rather than biorelevant performance or shelf life determination. Future work should therefore focus on targeted raw-material qualification, factorial formulation studies, pH-shift dissolution, direct matrix-structure measurements, nonlinear kinetic analysis, and longer ICH-compliant stability testing. Prior to commercial manufacturing scale-up, a stability-indicating HPLC assay should be fully validated to quantify the active pharmaceutical ingredient and relevant degradation products, and Powder X-Ray Diffraction (PXRD) analysis should be conducted to evaluate potential crystallographic changes associated with processing, storage, and manufacturing scale-up.

## 4. Conclusions

This study supports the potential of rice bran wax (RBW) as a natural lipid excipient for sustained release diclofenac sodium matrix tablets. Processing method and RBW concentration both influenced tablet properties and drug-release behavior, with melt granulation providing the greatest release retardation among the methods evaluated. SEM observations of the processed powders and granules suggested more extensive wax-mediated agglomeration following melt granulation; however, internal matrix continuity and homogeneity were not directly examined. FTIR and DSC provided no clear evidence of a major drug–excipient interaction and showed that a detectable crystalline fraction of diclofenac sodium remained after processing, although minor degradation could not be excluded. Increasing the RBW fraction while decreasing the SMCC fraction progressively slowed drug release, whereas 60% (*w*/*w*) RBW caused excessive retardation. Among the formulations tested, 55MG2, containing 55% (*w*/*w*) RBW and sintered at 80 °C for 2 h, met the dissolution limits specified in the USP–NF monograph for Diclofenac Sodium Extended-Release Tablets and showed close agreement with the commercial reference product. The preliminary 4-week accelerated study also showed no substantial deterioration in the measured physicochemical properties or dissolution profile, although a modest increase in drug release was observed. Overall, the findings indicate that melt granulation with RBW is a practical approach for developing hydrophobic sustained release matrix tablets and warrants further evaluation using additional drug candidates, longer stability studies, and direct characterization of matrix structure and release mechanisms.

## Figures and Tables

**Table 1:** Composition of tablet formulations with inversely varied RBW and SMCC contents *.

Composition	Amount per Tablet (mg)			
	45%	50%	55%	60%
Diclofenac Sodium (DFS)	100	100	100	100
Rice Bran Wax (RBW)	180	200	220	240
Silicified Microcrystalline Cellulose (SMCC)	118	98	78	58
Magnesium stearate (MGS)	2	2	2	2
Total	400	400	400	400

* RBW and SMCC were varied inversely to maintain a constant tablet weight. Therefore, the formulations represent a coupled increase in hydrophobic RBW and a decrease in relatively hydrophilic SMCC.

**Table 2:** Porosity, hardness, friability, and thickness of DFS tablets formulated using different methods *.

Formulation	%RBW	Porosity (%)	Hardness (N)	Friability (%)	Thickness (mm)
SM	45	13.63 ± 0.35 ^a^	88.1 ± 2.4 ^defg^	2.41	4.00 ± 0.02 ^hi^
	50	12.09 ± 0.27 ^b^	89.3 ± 1.9 ^def^	1.78	3.99 ± 0.01 ^hi^
	55	11.05 ± 0.23 ^c^	85.2 ± 3.2 ^fgh^	2.04	4.22 ± 0.01 ^de^
	60	10.73 ± 0.17 ^cd^	75.4 ± 4.2 ^jk^	3.57	4.50 ± 0.06 ^a^
DG	45	12.07 ± 0.16 ^b^	91.3 ± 7.0 ^cde^	1.18	3.96 ± 0.02 ^i^
	50	10.13 ± 0.49 ^cd^	95.4 ± 4.3 ^bc^	0.77	4.05 ± 0.02 ^g^
	55	10.62 ± 0.58 ^cd^	85.0 ± 2.2 ^fgh^	1.21	4.15 ± 0.02 ^f^
	60	10.23 ± 0.33 ^cd^	81.9 ± 2.7 ^hi^	0.89	4.48 ± 0.01 ^a^
ES	45	11.71 ± 0.25 ^b^	93.6 ± 2.9 ^cd^	0.78	3.89 ± 0.00 ^j^
	50	11.45 ± 0.33 ^b^	95.5 ± 2.1 ^bc^	0.52	4.23 ± 0.02 ^de^
	55	11.52 ± 0.46 ^b^	83.0 ± 4.0 ^ghi^	0.86	4.24 ± 0.01 ^cd^
	60	11.65 ± 0.18 ^b^	86.1 ± 2.0 ^efgh^	0.64	4.48 ± 0.01 ^a^
PMG	45	10.24 ± 0.16 ^cd^	100.4 ± 2.7 ^ab^	0.45	4.03 ± 0.02 ^gh^
	50	10.22 ± 0.51 ^cd^	95.1 ± 1.2 ^bc^	0.58	4.19 ± 0.01 ^ef^
	55	10.08 ± 0.09 ^cd^	78.5 ± 0.9 ^ij^	0.82	4.25 ± 0.01 ^cd^
	60	9.69 ± 0.34 ^d^	71.7 ± 5.9 ^kl^	0.61	4.30 ± 0.03 ^b^
MG	45	10.98 ± 0.36 ^c^	102.6 ± 2.5 ^a^	0.55	3.98 ± 0.03 ^i^
	50	10.59 ± 0.42 ^cd^	96.5 ± 1.1 ^bc^	0.67	4.23 ± 0.02 ^de^
	55	10.10 ± 0.09 ^cd^	83.3 ± 5.5 ^ghi^	0.82	4.25 ± 0.02 ^cd^
	60	10.20 ± 0.19 ^cd^	69.5 ± 3.0 ^l^	0.37	4.28 ± 0.06 ^bc^

* Values for porosity, hardness, and thickness are presented as mean ± SD. Within each parameter, values sharing at least one superscript letter are not significantly different according to one-way ANOVA followed by Tukey’s HSD test (p>0.05). Friability was determined from a single pooled-tablet test and was therefore not subjected to statistical analysis.

**Table 3:** Cumulative percentage of diclofenac sodium released from MG2 formulations and the commercial reference product compared with the USP–NF dissolution limits.

Formulation	Cumulative Percentage of Drug Release				
	1 h	5 h	10 h	16 h	24 h
45MG2	30.54 ± 2.07	75.18 ± 6.10	87.15 ± 5.68	92.17 ± 4.98	97.68 ± 5.89
50MG2	22.04 ± 2.72	70.21 ± 11.56	84.13 ± 3.50	87.65 ± 5.98	92.88 ± 4.53
55MG2	21.32 ± 7.79	63.59 ± 9.63	77.24 ± 7.36	82.52 ± 3.05	88.48 ± 7.78
60MG2	7.84 ± 1.00	21.73 ± 0.40	42.60 ± 4.16	65.84 ± 5.85	80.55 ± 9.34
Commercial reference	25.79 ± 1.75	56.85 ± 2.96	79.89 ± 3.35	91.55 ± 3.01	99.07 ± 5.88
USP range	15–35	45–65	65–85	75–95	80–100

Values are presented as mean ± SD. MG2 indicates melt-granulated formulations sintered at 80 °C for 2 h. The USP ranges correspond to the acceptance criteria for Diclofenac Sodium Extended-Release Tablets.

**Table 4:** Correlation coefficients (r^2^) of kinetic models and the release exponent (n) from the Korsmeyer–Peppas model describing drug release from diclofenac sodium–rice bran wax matrix tablets prepared by the optimized melt granulation method (MG2; 2 h sintering).

Kinetic Model/ Parameter	Formulation			
	45MG2	50MG2	55MG2	60MG2
Zero Order, r^2^	0.6797	0.6545	0.7070	0.9769
First Order, r^2^	0.9734	0.8980	0.9070	0.9910
Higuchi, r^2^	0.9683	0.9590	0.9788	0.8763
Korsmeyer–Peppas, r^2^	0.9203	0.9016	0.9232	0.9913
Korsmeyer–Peppas, n	0.3702	0.4594	0.4541	0.7549

Abbreviation: MG2 = melt granulation formulation sintered at 80 °C for 2 h. Note: The  n values were calculated from the slope of the regression of $log\left(M_{t}/M_{\infty }\right)$ against logt using five available sampling points. Because the fitting range extended beyond $M_{t}/M_{\infty }=0.60$ the values were not used for definitive mechanistic classification.

**Table 5:** Weibull model parameters for the dissolution profiles of the commercial product and 55MG2 tablets.

Sample	α (h)	β	$t_{50}$ (h)	RMSE	$R^{2}$
Commercial DFS	5.56 ± 0.72	0.79 ± 0.05	3.49 ± 0.38	3.77 ± 1.34	0.988 ± 0.007
55MG2–Week 0	6.64 ± 0.76	0.70 ± 0.07	3.91 ± 0.26	5.52 ± 2.21	0.967 ± 0.023
55MG2–Week 4	5.43 ± 0.87	0.77 ± 0.05	3.37 ± 0.42	1.78 ± 0.98	0.996 ± 0.004

Values are expressed as mean ± standard deviation from three independently fitted replicate dissolution profiles. The two-parameter Weibull model was fitted with asymptotic release fixed at 100%.

**Table 6:** Difference (*f*_1_) and Similarity (*f*_2_) Factors for Dissolution Profile Comparison.

	Difference Factor (f_1_)	Similarity Factor (f_2_)	Interpretation
55MG2-Wk0 vs. Control	7.74	58.62	Similar
55MG2-Wk4 vs. Control	4.66	70.62	Similar
55MG2-Wk4 vs. 55MG2-Wk0	6.81	65.40	Similar

For descriptive interpretation, *f*_1_ values of 0–15 and *f*_2_ values of 50–100 were considered indicative of close profile agreement. These values were not interpreted as formal regulatory evidence of dissolution equivalence.

## Data Availability

The original contributions presented in this study are included in the article. Further inquiries can be directed to the corresponding author.
